# Multi-dimensional factors of behavioral intention of COVID-19 booster vaccination among people having contracted COVID-19—a 7-city study conducted during the last national major outbreak in China

**DOI:** 10.1186/s12889-025-23915-6

**Published:** 2025-12-30

**Authors:** Xiaoying Zhang, Hui Lu, Yong Xu, Junqiang Ying, Xianying Wen, Lei Luo, Meng Wang, Muwen Liu, He Wang, Xingyi Geng, Xuchong Zhao, Biyu He, Tao Liu, Remina Maimaitijiang, Qian Yang, Yanqiu Yu, Joseph T. F. Lau

**Affiliations:** 1 College of Public Health, Shanghai University of Medicine and Health Sciences, Shanghai, 201318 China; 2 Oujiang Laboratory (Zhejiang Lab for Regenerative Medicine, Vision and Brain Health), Wenzhou Medical University, Wenzhou, China; 3 Neijiang Center for Disease Control and Prevention, Neijiang, China; 4 Mianyang Center for Disease Control and Prevention, Mianyang, China; 5 Hangzhou Center for Disease Control and Prevention, Hangzhou, China; 6 Ji’nan Center for Disease Control and Prevention, Ji’nan, China; 7 Shanghai Municipal Center for Health Promotion, Shanghai, China; 8 Community Health Service Center of Friendship Street, Baoshan District, Shanghai, China; 9 Department of Public Health, Shihezi University School of Medicine, Shihezi, China; 10 School of Public Health and the Department of Geriatrics of the Fourth Affiliated Hospital, Zhejiang University School of Medicine, Zhejiang University, Hangzhou, China; 11 School of Public Health, Fudan University, Shanghai, China; 12 Public Mental Health Center, School of Mental Health, Wenzhou Medical University, Wenzhou, China; 13 Zhejiang Provincial Clinical Research Center for Mental Disorders, The Affiliated Wenzhou Kangning Hospital, Wenzhou Medical University, Wenzhou, China; 14 Center for Health Behaviors Research, The Chinese University of Hong Kong, Hong Kong, China

**Keywords:** COVID-19, Booster vaccination, Illness representation, Cognitive factors, Emotional factors, China, Depression, Social support, Social distancing

## Abstract

**Introduction:**

The COVID-19 reinfection rate is high, and COVID-19 remains a significant public health threat. Booster vaccination is effective in reducing reinfection and the severity of infection. To increase the booster vaccination rate among people having contracted COVID-19 (PCC), it is warranted to understand the prevalence and factors of behavioral intention to take up COVID-19 booster vaccination. Multi-dimensional factors, including cognitive factors (cognitive factors of illness representation (IR) and perceived inevitability of infection), emotional factors (emotional factors of IR and panic), psychosocial factors (depressive symptoms and social support), and a behavioral factor (social distancing behavior), were examined for their association with behavioral intention to take up COVID-19 booster vaccination after six months since the COVID-19 diagnosis (BI-BCV).

**Methods:**

An anonymous cross-sectional online survey was conducted among the adult PCC population in seven cities located in eastern, southern, western, northern, and north-western China using stratified clustered sampling from December 27, 2022 through January 9, 2023, which is during China’s last national major outbreak.

**Results:**

A total of 5,757 PCC were included in the study. The prevalence of BI-BCV was 65.8%. Age, employment status, community type (rural/urban), chronic disease control status, marital status, education level, infection timing, and city were significantly associated with BI-BCV. The multivariate logistic regression yielded that IR constructs of treatment control, IR of illness coherence, social support, and social distancing behavior factors were positively associated with BI-BCV. In addition, IR of perceived consequences of COVID-19, perceived inevitability of infection, and depressive symptoms were negatively associated with BI-BCV. The emotional factors (panic and emotional IR) were non-significant.

**Conclusions:**

The BI-BCV prevalence was lower than ideal and warrants health promotion. We found that some cognitions (IR constructs of illness coherence, treatment control, and perceived consequences of COVID-19 and perceived inevitability of infection), behavior (social distancing), psychosocial factors (depression and social support), but not emotional states related to COVID-19, were potential determinants of BI-BCV. Future longitudinal studies are warranted to confirm the findings. Health workers may consider modifications of the significant factors found in this study.

**Supplementary Information:**

The online version contains supplementary material available at 10.1186/s12889-025-23915-6.

## Introduction

The first novel Coronavirus Disease 2019 (COVID-19) virus outbreak occurred in 2019. It then quickly spread all over the world. In early 2020, the World Health Organization (WHO) classified it as a Public Health Emergency of International Concern (PHEIC) event and then declared it as a pandemic [1]. All countries were hit during the recurring waves of COVID-19 outbreaks, characterized by surges in the number of cases and emergence of new variants (e.g., the Alpha, Delta, and Omicron variants and its subvariants) that involved different levels of infectivity and fatality [2–4]. As of October 2024, more than 776 million people worldwide have contracted COVID-19, resulting in more than seven million deaths [5]. At the societal level, the pandemic has caused severe economic damage and financial distress, partially due to extended lockdowns and business closures [6]. At the individual level, it has also profoundly affected multiple aspects of life, including physical health, education, social interaction, healthcare delivery, and mental health [7–11]. According to the World Health Organization, the prevalence of depression and anxiety has increased by 27.6% in the aftermath of the pandemic [12]; factors of mental distress included experience of infection, fear, loss of loved ones, and quarantine measures [13, 14]. Although COVID-19 is no longer a pandemic, the reinfection rate had been as high as 28% when the BQ.1/BQ.1.1 variants were involved [15]. COVID-19 reinfection increased the risks of death, hospitalization, long COVID, and mental distress [16]. COVID-19 thus remains a significant public health threat [17], which might have gradually been overlooked.

A meta-analysis of 91 published studies reported the effectiveness of COVID-19 vaccination in reducing reinfection [18]. As natural immunity wanes over time and new variants keep evolving, vaccination among people having contracted COVID-19 (PCC) is critically important. Booster vaccination is effective in reducing reinfection and the severity of infection [19, 20]. The World Health Organization and some country authorities recommend regular COVID-19 vaccination six months since the diagnosis [21–23]. However, previous studies suggest that PCC have a lower intention to receive booster doses of vaccine compared to the general population [24, 25]. To design effective interventions, it is warranted to understand the prevalence and factors potentially influencing behavioral intention to take up COVID-19 booster vaccination after six months since the COVID-19 diagnosis (BI-BCV) among PCC. Such factors can be multi-dimensional and may include cognitive factors, behavioral factors, emotional factors, and psychosocial factors.

Cognitive factors are important in determining BI-BCV. First, how people feel and think about COVID-19, a concept known as illness representation (IR), may affect COVID-19 vaccination [26]. IR comprises cognitive representations and emotional representations. In response to a stressor due to an illness, according to the Common-Sense Model of Illness [27], individuals would develop cognitive and emotional representations in two parallel processes, both of which would affect coping and outcome appraisal that determine health-related outcomes [28]. Cognitive illness representations include consequences (assessing the severity of the illness), timeline chronic (assessing the time frame of disease development, duration, and recovery), control (considering whether the illness is under control), identity (labeling the illness and identifying its symptoms), illness coherence (overall comprehensibility of the illness), and cause (attributing likely causes of the illness). Cognitive IR was associated with influenza vaccination intention [29] and COVID-19 vaccination intention [26]. Importantly, IR is modifiable through interventions [30].

Apart from IR, perceived susceptibility is another important cognitive factor that was significantly associated with COVID-19 preventive behaviors in general [31, 32] and vaccination behavior or intention in particular [33, 34]. Perceived susceptibility is one of the constructs of the Health Belief Model [35]. The highest level of perceived susceptibility is represented by the perceived inevitability of contracting COVID-19. During the explosive phase of the COVID-19 pandemic in China, about 250 million people were infected within a remarkably short period of two weeks [36]. Given this context, it is reasonable to assume that some individuals might have perceived the virus was extremely infectious [37], to the extent that no measure would be able to prevent transmissions, a proportion of the public would then perceive COVID-19 infection as inevitable. Logically, such a perception would defer vaccination decision. The association between perceived inevitability and COVID-19 vaccination had not been examined prior to this study.

Emotional factors of BI-BCV are also important. As seen, emotional illness representations of COVID-19 (negative emotional responses to the illness including worry and anger) were associated with lower levels of COVID-19 vaccination behavior/intention [38]. According to the emotion regulation theory, negative emotions would result in emotion dysregulation, which would in turn reduce adaptive actions (e.g., vaccination) [39], implying that negative emotions would be associated with lower vaccination intention. In contrast, some previous studies reported a positive association between worries related to COVID-19 and vaccination intention [40]. It is plausible that individuals would be motivated to take action to reduce the worries [41]. The results are hence mixed and require further investigations.

The third dimension of the factors of BI-BCV included psychosocial factors. The pandemic caused tremendous mental distress, such as increase in depression incidence and prevalence [12]. For instance, the WHO declared that global depression increased by 27.6% during the pandemic [12]. Depression was significantly and negatively associated with COVID-19 vaccination behavior and intention [42, 43], as mental distress and related self-stigma may compromise help-seeking behaviors [44]. In contrast, social support played an important role in overcoming difficulties encountered during the pandemic [45, 46]; it was positively associated with both preventive behaviors in general [47–49] and COVID-19 vaccination in particular [50, 51].

The fourth dimension of BI-BCV factors included the practice of preventive behavior (social distancing). Social distancing was one of the most important COVID-19 preventive measures [52–54] strongly emphasized by the government and the public in China [55]. From one perspective, since social distancing and COVID-19 vaccination are both preventive measures against COVID-19 infection, it is expected that they would be positively correlated with each other, as reported in some previous studies [56, 57]. From another angle, the risk compensation theory [58] posits that the adoption of one protective behavior (e.g., social distancing) would reduce motivation to adopt other measures (e.g., vaccination) because of the lowered risk of infection. Thus, individuals practicing strict social distancing might regard COVID-19 vaccination as less necessary. Hence, the relationship between social distancing and COVID-19 vaccination remains unclear. Moreover, no study has yet explored such an association among PCC. This study aims to address this gap in the literature.

Contextually, the present study was conducted in seven Chinese cities. China consistently exercised strict control measures against COVID-19 from February 2020 to December 2022 (e.g., quarantine for inbound travelers, closure of public venues, compulsory testing of residents living in building blocks where infections were found, and working from home). In December 2022, given the milder harm of the variants, the high vaccination rate, and the readiness of the medical system, the government removed all COVID-19 control measures within a very short notice [59]. The sudden termination of the control measures in China resulted in an unprecedentedly rapid spread of the virus across the country [60]. This study is a unique one as it collected data during this critical period of health policy change and unprecedented outbreak.

The present study investigated the prevalence and factors of BI-BCV among Chinese adult PCC who have taken up at least one dose of the COVID-19 vaccine. Since the majority of the population has ever been vaccinated [61], it is implicative to understand factors of BI-BCV in the ever-vaccinated group. Cognitive factors (the cognitive IR constructs and perceived inevitability of infection), emotional factors (IR emotional representation and panic related to COVID-19), psychosocial factors (social support and depressive symptoms), and a behavioral factor (social distancing behavior) were examined. It is hypothesized that some of the cognitive IR constructs (consequences, timeline chronic, control, identity, and illness coherence), perceived inevitability of infection, and social support would be positively associated with BI-BCV, while depressive symptoms would be negatively associated with BI-BCV. Two-sided alternative hypotheses were set up for some emotional factors (the IR constructs of emotional representation and illness concern, and panic) and social distancing, because as mentioned, both positive and negative associations are plausible and have been reported in previous studies [25, 40, 56, 62]. Most of such factors of BI-BCV have not been investigated among PCC. There is also a dearth of studies looking at booster vaccination among PCC. Since the abovementioned factors are modifiable, the finding of this study has implications for improving COVID-19 vaccination among PCC.

## Materials and Methods

### Study design and data collection

From December 27, 2022 to January 9, 2023, an anonymous large-scale cross-sectional online survey was conducted among the adult PCC population in seven cities located in eastern (Hangzhou and Shanghai), southern (Guangzhou), western (Neijiang and Mianyang), northern (Ji’nan), and north-western (Shihezi) China.

The inclusion criteria were: (1) age 18–60 and (2) COVID-19 diagnosis received from December 1, 2022 till the survey date. Since the Chinese government started loosening some COVID-19 control measures in December 2022, those whose COVID-19 infection was diagnosed prior to December 1, 2022 (i.e., before the policy change and the severe outbreak) were excluded from data analysis to maintain homogeneity. Those not having taken up any dose of COVID-19 vaccination were also excluded, as this study focused on booster vaccination whose determinants might be different from those of the intention of first-dose COVID-19 vaccination. Furthermore, those never vaccinated were only a minority since over 90% of the Chinese population had taken up at least one dose of COVID-19 vaccination as of June 2022 [61].

Stratified clustered sampling was performed. Chinese cities are typically divided into several districts (counties), within which there are several ‘sub-districts’ comprising a number of ‘communities’. A ‘community’ may have clusters of hundreds to thousands of residents [63]. During the pandemic, many ‘communities’ have established WeChat groups to facilitate communications between the local Center for Disease Control and Prevention (abbreviated as CDC in the following text) and the residents about COVID-19 matters (e.g., free mass testing and health promotion). WeChat is a widely used Chinese multi-functional app that integrates messaging, social media, and mobile payment services, with over a billion active users [64]. Such WeChat groups covered most of the community’s households.

Coordinated by community leaders (e.g., resident representatives and staff of the local CDCs), three districts were firstly selected from each city by convenience sampling, and a sub-district was chosen from each district (stratum). At least three ‘communities’ (clusters) were selected from each sub-district. Invitations were sent to all WeChat group members of the selected communities. One participant from each household was invited to self-administer the online questionnaire through the Wenjuanxing system, a professional online survey platform widely used in China. No names nor any identifiable information were collected. Participants were explained in the invitation message about the purpose of the survey, its anonymity, and that the information would be used by only the research team; they were told that refusal would not affect them in any way. Participants were requested to provide their informed consent prior to administering the questionnaire. No incentives were given to the participants. Ethics approval was granted by the ethics committee of the School of Public Health, Zhejiang University (No. ZLG202301-01). Although a national representative sample was not feasible, the design of the present study tried to include cities in different regions of the country. Being well supported by local institutions having good relationships with the residents, the study attempted to increase representativeness as much as possible by using stratified cluster sampling. As all the communities had formed WeChat groups during the pandemic, it was a feasible study design.

A quality check was exercised. For instance, those who spent an extremely short time completing the online questionnaire and those who showed missing values in > 20% of all the question items were removed from data analysis. A sample size of 5000 was used for planning; it would limit the obtained 95% confidence interval for the prevalence of BI-BCV to be narrower than plus or minus 1.39%.

### Measures

The questionnaire was developed by a panel of public health researchers experienced in behavioral health and COVID-19 prevention research and/or vaccination research; all of them have published multiple papers in those areas. The design of the questionnaire was based on an extensive literature review. All except one of the scales used in the present study have been validated and/or been used in previous studies. As Chinese versions of those scales were available, no translation was necessary. The exception was the perceived inevitability of infection, which was created in this study as no study has investigated this construct. The variable has special relevance as the survey was conducted during the explosive phase of the pandemic in China, during which an unprecedented outbreak infected a major proportion of the national population within several weeks, including those having received vaccination [36]. It is hence plausible that some people might perceive an inevitability of infection during the study period.

#### Background factors

Background information was collected, including age, sex, current marital status (married or else), employment status (fulltime employment or else), education levels (high school or lower, undergraduate or above), community type (urban or rural), the timing of COVID-19 diagnosis, chronic disease status, and the city where the survey took place.

#### Behavioral intention of COVID-19 booster vaccination after six months from now (BI-BCV)

It was assessed by the question: “How likely do you plan to take up the COVID-19 vaccine after six months from now?” The 6-month period was set as PCC were recommended to take up COVID-19 vaccines after but not within six months since the diagnosis [21–23]; the criterion hence ensured that all participants were recommended to take up the vaccines at the time of the survey. The Likert scale responses were definitely not [1], unlikely [2], half-half [3], likely [4], and definitely [5]. The scale has been used in several previous COVID-19 studies conducted in China [65, 66]. Like these studies [67, 68], a binary variable was formed by recoding 4 and 5 (affirmative) as “1” and else as “0” (non-affirmative).

#### Cognitive factors

*Cognitive IR of COVID-19.* The six cognitive items of the Brief Illness Perception Questionnaire for COVID-19 (B-IPQ-COVID-19) (11-point Likert scale) were used in this study [69]. The domain of consequences was assessed by “How much has the COVID-19 infection affected your life?” (0 = does not affect at all to 10 = severely affects my life), timeline chronic by “How long do you think your COVID-19 condition would last?” (0 = a very short time to 10 = forever), personal control by “How much control do you have over your COVID-19 conditions?” (0 = absolutely no control to 10 = extreme amount of control), treatment control by “How much do you think treatment can help your COVID-19 conditions?” (0 = not at all to 10 = extremely helpful), identity by “How many symptoms related to COVID-19 have you experienced?” (0 = no symptoms at all to 10 = many severe symptoms), illness coherence by “How well do you feel you understand COVID-19?” (0 = not understand at all to 10 = understand very well. The validated version of B-IPQ was used [69]. Previous studies have used this scale for assessing illness representation of COVID-19 [69, 70].

*Perceived inevitability of infection.* The construct was measured by the question, “I would be unable to protect myself from contracting/re-contracting COVID-19, no matter what protection measures I am going to take up.” The participants rated their responses on a Likert scale from 1 (strongly disagree) to 5 (strongly agree). A higher score indicated stronger perceived inevitability. The item was created for this study as no previous study has looked at the association between this construct and COVID-19 behaviors.

#### Emotional factors

*Emotional representation of IR.* It was assessed by two questions of the B-IPQ-COVID-19. Emotional representation was assessed by “How much have your COVID-19 conditions affected you emotionally (e.g., make you angry, scared, upset, or depressed)?” (0 = not at all to 10 = extremely) and illness concern by “How concerned are you about your COVID-19 conditions?” (0 = not at all concerned to 10 = extremely concerned). The tool has been validated [69] and the Chinese version has been used in previous studies [71], including some COVID-19 studies [72].

*Panic*. Panic was assessed by counting the number of conditions: (1) panic about older people or children in the participant’s family being infected or re-infected with COVID-19, (2) lack of medicine for COVID-19 or antigen testing kits, and (3) the COVID-19 situation in the city the participant was living in. Each item was measured by a binary question (0/1). The former two items have been used in some previous studies [73–75] including Chinese studies [76]. The value of the count variable ranged from 0 to 3.

#### Psychosocial factors

*Social support*. It was formed by adding up the three item scores reflecting emotional support, instrumental support, and support related to obtaining COVID-19 medicine or antigen testing kits. An example question was: “Currently if you need someone to talk to or for emotional support, your family and friends will be there for you”. The participants rated on a seven-point Likert scale from 1 (strongly disagree) to 7 (strongly agree). The overall score ranged from 3 to 21. A higher score indicated a high level of social support. The Cronbach’s alpha of the scale was 0.86. Similar scales have been used in a number of published studies conducted in China [77], including COVID-19 studies [78].

*Depression.* The 9-item Patient Health Questionnaire (PHQ-9) was used to evaluate the level of depressive symptoms [79]. The items were rated on 4-point Likert scales (0 = not at all to 3 = nearly every day). The validated Chinese version was used [80]. The total scale score ranged from 0 to 27. A higher score indicated more severe depressive symptoms. Total scores of 5, 10, 15, and 20 represent cut-off points for mild, moderate, moderately severe, and severe depression, respectively. The validity and reliability of the Chinese version have been established in previous studies [80]. The Cronbach’s alpha of the scale in this study was 0.93. It has been used in many COVID-19 studies conducted in China [66, 81].

#### Behavioral factor

*The level of social distancing behaviors* was measured by the modified five-item scale: “In the past week, how often did you do the following: 1) not going out unless necessary, 2) avoiding attending social gatherings, 3) reducing meeting and contacting people you know, 4) avoiding going to crowded places, and 5) avoiding taking public transportation?”. The scale was developed in Chinese and was validated in China [82]. The rating of the Likert scales was 1 (never) to 5 (always). A higher scale score indicated a higher level of social distancing behavior. The Cronbach’s alpha of the scale in this study was 0.91.

#### Statistical analysis

Descriptive statistics (e.g., mean, standard deviation, range, and proportion) were derived and presented in the manuscript. As the dependent variable (BI-BCV) was a binary variable, logistic regression was the appropriate method for statistical analysis. Univariable logistic regression analyses were firstly conducted to test the significance of the associations between each of the background variable/independent variable and BI-BCV; crude odds ratio (ORc) and respective 95% CI were derived. The second step of statistical analysis involved multiple logistic regression analysis which adjusted for the background variables measured in this study, as such background variables could be potential confounders. The multiple logistic regression analysis was also conducted for each of the independent variables. Again, the adjusted odds ratio (ORa), and their respective 95% confidence intervals (CIs) were derived. Statistical significance was defined as a two-tailed *p*-value < 0.05. Data analysis was conducted by using Stata 17.0.

## Results

### Participants’ characteristics

As shown in Table 1, a majority of the participants were female (69.8%), lived in urban areas (82.6%), had received college or above education (77.3%), had a fulltime job (73.1%), and were currently married (75.5%). About half of them aged 31–45 years (48.4%); 83.4% did not have chronic diseases. Hangzhou had the highest number of participants (*n* = 1821, 31.6%). More than two-thirds (68.9%) of the participants were diagnosed between December 15 and 28, 2022 (Dec 1–7: 2.0%; Dec 8–14: 10.7%; Dec 15–21: 33.9%; Dec 22–28: 35.0%; Dec 29–Jan 9: 18.4%). The prevalence of BI-BCV was 65.8%.

**Table 1:** Descriptive statistics (*n* = 5,757)

	*n*	%
City		
Hangzhou	1821	31.6
Shanghai	231	4.0
Ji’nan	660	11.5
Neijiang	1284	22.3
Mianyang	1174	20.4
Guangzhou	411	7.1
Shihezi	176	3.1
Age group (years)		
18–30	1616	28.1
31–45	2784	48.4
46–60	1357	23.6
Sex		
Male	1738	30.2
Female	4019	69.8
Education level		
High school or lower	1309	22.7
College or above	4448	77.3
Employment status		
Fulltime	4210	73.1
Not fulltime (part-time or not employed)	1547	26.9
Current marital status		
Married	4344	75.5
Not married	1413	24.5
Chronic disease status		
No	4802	83.4
Yes, poorly controlled	259	4.5
Yes, well controlled	696	12.1
Community type		
Urban	4757	82.6
Rural	1000	17.4
Timing of COVID-19 diagnosis		
Dec 1 to Dec 7	114	2.0
Dec 8 to Dec 14	613	10.7
Dec 15 to Dec 21	1948	33.9
Dec 22 to Dec 28	2015	35.0
Dec 29 to Jan 9	1067	18.4
Behavioral intention of COVID-19 booster vaccination after six months from now (BI-BCV)		
No	1969	34.2
Yes	3788	65.8

The results are presented in Table 2. The mean values (standard deviation [SD], range) of the IPQ items ranged from 5.30 to 6.10 (range = 0–10). The mean values were 3.50 (SD = 1.06, range = 1–5) for perceived inevitability of infection, 1.45 (SD = 1.01, range = 0–3) for panic, 16.02 (SD = 3.58, range = 3–21) for social support, 9.64 (range 0–27, SD = 6.56) for PHQ-9, and 19.09 (SD = 5.20, range = 5–25) for social distancing behavior. The prevalence of mild or above depression and moderate or above depression was 76.9% and 41.1%, respectively. About three-quarters (74.2%) of the participants felt panic about infected children or older adults in the family; 38.7% felt panic about the lack of medicine or antigen testing kits; 32.5% felt panic about their city’s COVID-19 situation. The frequency of the count variable for panic was 18.4%, 38.5%, 22.4%, and 20.7% for the count score of 0, 1, 2, and 3, respectively.

**Table 2:** Descriptive statistics of the multidimensional independent variables (*n* = 5,757)

Dimensions	Variables	Range	Mean/number (SD/percentage)
Cognitive factors	IPQ-cognitive factors		
	Consequences	0–10	6.55 (2.55)
	Timeline chronic	0–10	5.90 (2.30)
	Personal control	0–10	5.62 (2.36)
	Treatment control	0–10	5.64 (2.38)
	Identity	0–10	5.70 (2.09)
	Illness coherence	0–10	6.10 (2.20)
	Perceived inevitability of infection	1–5	3.50 (1.06)
Emotional factors	IPQ- emotional factors		
	Illness concern	0–10	5.97 (2.63)
	Emotional representation	0–10	5.30 (2.59)
	Panic	0–3	1.45 (1.01)
	0		1059 (18.4%)
	1		2216 (38.5%)
	2		1291 (22.4%)
	3		1191 (20.7%)
Psychosocial factors	Social support	3–21	16.02 (3.58)
	Depressive symptoms (PHQ-9)	0–27	9.64 (6.56)
	No depressive symptoms (0–4)		1332 (23.1%)
	Mild (5–9)		2057 (35.7%)
	Moderate (10–14)		1050 (18.2%)
	Moderately-severe (15–19)		889 (15.4%)
	Severe (20–27)		429 (7.5%)
Behavioral factor	Social distancing behavior	5–25	19.09(5.20)

### Background factors of BI-BCV

The results are shown in Table 3. Background factors positively associated with BI-BCV included age (reference group = 18–30; age 31–45: ORc = 1.40, 95% CI: 1.23, 1.59; age 46–60: ORc = 2.11, 95% CI: 1.80, 2.46), not fulltime employment (reference = fulltime employment: ORc = 1.17, 95% CI: 1.04, 1.33), rural community type (ORc = 1.36, 95% CI: 1.17, 1.58), well-controlled chronic disease status (reference group = no chronic diseases; ORc = 1.29, 95% CI: 1.08, 2.03). Background factors that were negatively associated with BI-BCV included currently not married (reference = currently married, ORc = 0.73, 95% CI: 0.65, 0.83), college or higher education level (reference = high school or lower; ORc = 0.76, 95% CI: 0.68, 0.85), and poorly controlled chronic disease status (reference group = no chronic diseases; ORc = 0.61, 95% CI: 0.47, 0.78). In addition, using those infected during the first week of December (December 1 to December 7) as the reference group, those infected during the fifth or sixth weeks (December 29 to January 9) were less likely than others to show BI-BCV (ORc = 0.59, 95% CI: 0.38, 0.90). Last, using Hangzhou participants as the reference group, participants in Shanghai showed significantly lower BI-BCV, and those in Ji’nan, Neijiang, Mianyang, and Guangzhou showed significantly higher BI-BCV (refer to Table 3 for detailed information).

**Table 3:** Background factors of BI-BCV (*n* = 5,757)

	ORc (95% CI)	*p*
City		
Hangzhou	Reference = 1.0	
Shanghai	0.53 (0.40, 0.70)	< 0.001
Ji’nan	1.84 (1.52, 2.24)	< 0.001
Neijiang	1.19 (1.03, 1.39)	< 0.05
Mianyang	2.24 (1.90. 2.64)	< 0.001
Guangzhou	1.83 (1.45, 2.32)	< 0.001
Shihezi	1.20 (0.87, 1.65)	0.26
Age group		
18–30	Reference = 1.0	
31–45	1.40 (1.23, 1.59)	< 0.001
46–60	2.11 (1.80, 2.46)	< 0.001
Sex		
Female Male	Reference = 1.0 1.04 (0.92, 1.17)	0.49
Education level		
Higher school or lower	Reference = 1.0	
College or above	0.76 (0.68, 0.85)	< 0.001
Employment status		
Full time	Reference = 1	
Not full time	1.17 (1.04, 1.33)	< 0.05
Current marital status		
Married	Reference = 1	
Not married	0.73 (0.65, 0.83)	< 0.001
Community type		
Urban	Reference = 1	
Rural	1.36 (1.17, 1.58)	< 0.001
Time of COVID-19 diagnosis		
Dec1 to Dec 7	Reference = 1	
Dec 8 to Dec 14	0.98 (0.63, 1.52)	0.92
Dec 15 to Dec 21	0.80 (0.53, 1.22)	0.30
Dec 22 to Dec 28	0.74 (0.49, 1.12)	0.16
Dec 29 to Jan 9	0.59 (0.38, 0.90)	< 0.05
Chronic disease status		
No	Reference = 1.0	
Yes, poorly control	0.61 (0.47, 0.78)	< 0.001
Yes, well control	1.29 (1.08, 2.03)	< 0.01

*ORc* Crude odds ratio, *CI* Confidence interval

### Multi-dimensional factors of BI-BCV

The multivariable adjusted logistic regression results are shown in Table 4. The model was adjusted for the potential confounders of city, age, sex, education level, marital status, employment status, community type, chronic disease status, and timing of diagnosis. Factors positively associated with BI-BCV included the cognitive IR constructs of treatment control (ORa = 1.08, 95% CI: 1.05, 1.11), the cognitive IR of illness coherence (ORa = 1.04, 95% CI: 1.01, 1.07), social support (ORa = 1.04, 95% CI: 1.03, 1.06), and social distancing behavior (ORa = 1.01, 95% CI: 1.00, 1.03). Factors that were negatively associated with BI-BCV included the cognitive IR of severe perceived consequences of COVID-19 (ORa = 0.96, 95% CI: 0.93, 0.99), perceived inevitability of infection (ORa = 0.91; 95% CI: 0.86, 0.96), and depressive symptoms (ORa = 0.97, 95% CI: 0.96, 0.98). The cognition IR variables of timeline chronic (ORa = 1.02, 95% CI: 0.99, 1.05), personal control (ORa = 0.98, 95% CI: 0.96, 1.01), and identity (ORa = 0.97, 95% CI: 0.93, 1.01), the emotional IR variables of illness concern (ORa = 0.99, 95% CI: 0.96, 1.03) and emotional representation (ORa = 1.00, 95% CI: 0.97, 1.04), and the panic count variable (reference: no panic, ORa = 1.03, 95% CI: 0.97, 1.10) were non-significant.

**Table 4:** Multi-dimensional factors of BI-BCV (*n* = 5757)

Variables	ORc (95% CI)	*p*	ORa (95% CI)	*p*
Cognitive factors				
IPQ-cognitive factors				
Consequences	0.93 (0.91, 0.95)	< 0.001	0.96 (0.93, 0.99)	0.01
Timeline chronic	0.97 (0.85, 0.99)	< 0.05	1.02 (0.99, 1.05)	0.23
Personal control	1.05 (1.03, 1.08)	< 0.001	0.98 (0.96, 1.01)	0.24
Treatment control	1.09 (1.06, 1.12)	< 0.001	1.08 (1.05, 1.11)	< 0.001
Identity	0.94 (0.91, 0.96)	< 0.001	0.97 (0.93, 1.01)	0.16
Illness coherence	1.08 (1.05, 1.11)	< 0.001	1.04 (1.01, 1.07)	0.01
Perceived inevitability of infection	0.88 (0.83, 0.92)	< 0.001	0.91 (0.86, 0.96)	< 0.01
Emotional factors				
IPQ-emotional factors				
Illness concern	0.95 (0,93, 0,97)	< 0.001	0.99 (0.96, 1.03)	0.75
Emotional representation	0.94 (0.92, 0.96)	< 0.001	1.00 (0.97, 1.04)	0.79
Panic	0.95 (0.90, 1.01)	0.09	1.03 (0.97, 1.10)	0.29
Psychosocial factors				
Social support	1.06 (1.04, 1.07)	< 0.001	1.04 (1.03, 1.06)	< 0.001
Depressive symptoms	0.96 (0.95, 0.97)	< 0.001	0.97 (0.96, 0.98)	< 0.001
Behavioral factor				
Social distancing behavior	1.01 (1.00, 1.02)	< 0.05	1.01 (1.00, 1.03)	0.02

*ORc *Crude odds ratio, *CI* Confidence interval, *ORa* Adjusted odds ratio

## Discussion

Despite the authoritative recommendation, only about two thirds (65.8%) of the participants indicated BI-BCV after six months from the survey date. This prevalence was much lower than ideal and was lower than the prevalence of COVID-19 vaccination intention recorded in some general populations [24, 83]. However, such data were not comparable across studies as different social contexts (e.g., countries, phase of the pandemic, whether including only PCC) and tools were involved. A systematic review of 48 international studies reported that the pooled COVID-19 booster vaccination intention was 79%, ranging from 23 to 97% [24]. One study conducted in Hong Kong in August, 2022 reported that the intention of getting a booster dose of the COVID-19 vaccine among PCC was 59% [25]. Given the high reinfection rate, the findings confirm the urgent need for health promotion to increase booster vaccination among PCC.

Corroborating previous studies, the prevalence of BI-BCV increased with age [84, 85] and decreased with education level [86]. Older people may be more concerned about the harms of COVID-19 infection [87]. It is plausible that less educated individuals might overestimate the benefit of COVID-19 vaccination, and better educated participants might underestimate the safety of the vaccine [86]. Like some other studies [25], full-time employment was associated with lower BI-BCV. Those with full-time employment tended to be younger and might have less free time to take up vaccination [25]. Also consistent with other studies [88, 89], married people showed higher prevalence of BI-BCV, plausibly because they might worry more about infecting their spouse and children [88, 89]. Participants living in rural areas showed higher BI-BCV than their counterparts, plausibly because medical facilities are less developed in rural China than urban China [90], so rural residents might have stronger concerns about treatment in their rural residential setting and were more keen to attain stronger protection via vaccination. Furthermore, diagnosis made during the earlier part of the survey was associated with higher BI-BCV. It is plausible that those with earlier diagnosis had experiences associated with less well-organized medical services, lower preparedness, and hence higher levels of uncertainties and stress that would urge them to take up the vaccines.

Previous studies reported that chronic disease status was associated with higher vaccination intention [91, 92] but did not take into account whether the chronic conditions were well controlled for. The findings suggest that poorly controlled chronic disease status was negatively associated with BI-BCV, while well controlled chronic disease status was positively associated with BI-BCV. It is plausible that those with poorly controlled chronic diseases might have poorer health literacy, self-care behaviors, and self-efficacy in taking up self-care measures than their counterparts [93]; low levels of such attributes may diminish COVID-19 vaccination intention. In contrast, those with well-controlled chronic disease conditions tended to exercise good self-care and adopt preventive measures in general, leading to higher BI-BCV levels.

Two of the cognitive IR constructs were positively associated with BI-BCV. First, the positive association between perceived treatment control and BI-BCV corroborated other seasonal influenza vaccination [29] and pneumonia vaccination acceptance studies [94]. Those with stronger treatment control IR might have stronger trust in scientific development in general and believed that the COVID-19 vaccines were efficacious. Second, a positive association between illness coherence (i.e., understanding of COVID-19) and BI-BCV was found. This finding was similar to those related to influenza A/H1N1 and HPV vaccination intention [95, 96]. Knowledge about COVID-19, including its infectivity, consequences, long COVID, and efficacy of vaccines in prevention, were positively associated with vaccination intention [97–99]. In contrast to previous findings [94] and not supporting the initial hypothesis, a negative association was found between the cognitive IR of consequences and BI-BCV. A plausibility is that those who had suffered from severe symptoms, despite previous vaccination, no longer trusted the efficacy of the vaccines and would not indicate BI-BCV. These findings imply that health workers could consider implementing interventions to modify some cognitive IR constructs related to COVID-19 among ever-vaccinated PCC through interventions. Further research is needed to explore whether such associations would have been maintained in the post-pandemic stage.

Perceived inevitability of infection was negatively associated with BI-BCV. Although perceived susceptibility was positively associated with BI-BCV [100], given that perceived inevitability reflects an extremely high level of perceived susceptibility, some individuals might believe that given the virus’s extremely high infectivity, vaccination was no longer protective. Perceived inevitability would then be negatively associated with BI-BCV. It is acknowledged that this explanation requires further confirmation, as data on perceived vaccine efficacy during the study period were unavailable. This finding underscores the need for governments and public health authorities to convince PCC that COVID-19 reinfection is preventable and transmission is not inevitable. Additionally, the strong evidence about the efficacy of vaccination in preventing chance and severity reinfection should be disseminated to the PCC population and the general public to bolster confidence in vaccination efficacy.

The findings on two emotional factors are unexpected. Panic was not significantly associated with BI-BCV in the adjusted analysis. It is plausible that panic may lead to two potential consequences that have opposite impacts on BI-BCV. First, panic might result in maladaptive emotional responses [13], which might deprive people of motivation for self-protection [101]. The other side of the coin is that fear may create a motivation for individuals to remove the fear. According to the fear appeal theory, fear may result in a ‘fight’ response (taking action to minimize the fear) or a flight response (avoiding the fear) [102]. The ‘fight’ response would increase BI-BCV and may plausibly explain the observed positive association. Interestingly, the two emotional IR constructs of concern and emotional representation were also statistically non-significant. The overall pictures seem to suggest that emotional factors were not associated with booster vaccination among PCC, which contrasts against significant associations reported in studies looking at vaccinations regarding the first dose or completion of the primary series conducted in general populations [40, 70]. Future studies should investigate potential mediators in the association between emotional factors and COVID-19 vaccination intention (e.g., the maladaptive response effect versus the fight response).

Regarding the psychosocial behaviors, like in some previous studies, depression was negatively associated with BI-BCV [42, 43]. Depressed individuals tended to report stronger concern about the efficacy, side effects, and cost of vaccination and possessed a lower level of trust toward the government [43]; these attributes do not favor vaccination. As there was a 27.6% increase in the prevalence of depressive disorders globally during the COVID-19 pandemic [12], booster vaccination promotion should target those showing mental distress. As expected, social support was positively associated with BI-BCV, corroborating previous studies [51, 103]. Social support may enhance active coping resources [104] and reduce emotional and instrumental barriers against vaccination [105]. Furthermore, the Theory of Planned Behavior (TPB) postulates that subjective norm, defined as support being provided by significant others to perform the behavior, is a significant determinant of intention to perform the behavior; the two other factors of the TPB are attitudes and perceived behavioral control [106].

Corroborating previous studies [56, 107], we found that the behavioral factor of social distancing behavior was positively associated with BI-BCV. It is plausibly because both social distancing and vaccination were protective against COVID-19 transmission [108] and many people tend to use multiple means for COVID-19 prevention [109]. There are also findings that vaccinated people tend to comply less with social distancing [110]. According to the risk compensation theory [58], it is thus plausible that some people with strict social distancing believed that they did not need vaccination as they were ‘safe’. The findings of this study, however, do not support this hypothesis, adding to the understanding of the association between social distancing and vaccination. Future studies may analyze the mediation between social distancing and vaccination via perceived susceptibility of infection, which is beyond the scope of this study.

The present study has several unique strengths. It was conducted during a pandemic phase when the prevalence of COVID-19 increased exponentially. Interestingly, some new factors (e.g., perceived inevitability) emerged with interesting results; other factors’ associations with BI-BV were different from those obtained from studies conducted in other phases of the pandemic in and outside China. Comparisons, however, need caution. There is a lack of studies focusing on vaccinated PCC, whose vaccination behavior may differ from that of individuals without COVID-19 infection and/or vaccination.

The study has both theoretical and practical implications. First, as demonstrated, the factors influencing BI-BCV among PCC are multidimensional, encompassing cognitive, emotional, psychosocial, and behavioral components. No single theory fully captures the complexity of these factors. Instead, various theoretical frameworks—such as the Health Belief Model, Theory of Planned Behavior, Common Sense Model, Emotion Regulation Model, and Fear Appeal Theory—provide insights into specific aspects of the findings. Therefore, integrating these theories may offer a more comprehensive understanding of the multifaceted influences on vaccination behavior. Second, the negative association between perceived inevitability of infection and BI-BCV despite the commonly found positive association between perceived susceptibility and vaccination posts an interesting research on whether the association between perceived susceptibility and vaccination could be non-linear, and even be U-shaped. This implicative contention needs future research. Notably, there are some practical implications. First, some groups (e.g., currently unmarried individuals) showed a lower prevalence of BI-BCV and required attention in health promotion. Second, the factors of BI-BCV among vaccinated PCC may be different from those of first vaccination and/or those among non-infected individuals. Health promotion improving vaccination needs to take such contexts into account and be informed by research. Third, the present study reminds readers that vaccination studies for future pandemics need to consider phases of the pandemic and vaccination and infection status of the participants, as well as being cautious when comparing findings across COVID-19 vaccination studies. Lastly, health workers may try to modify some of the observed significant factors, such as those related to cognition and social support among PCC.

The present study also has some limitations. First, this was a cross-sectional study. Causal inferences are not feasible. Longitudinal studies are warranted to confirm the findings but was not feasible under the very fast changing post-survey situations. Second, vaccination is a socially desirable behavior; the level of BI-BCV might have been over-reported (i.e., a reporting bias). Third, the stratified clustered sampling procedure was not strictly implemented; it was not fully representative despite attempts made to enhance representativeness. Selection bias may occur (e.g., over-sampling older and better educated people, and only included people using WeChat). It was not feasible to record the response rate. Fourth, although the survey collected data from seven cities in different regions in China, it was not a nationally representative sample; caution is needed when generalizing the findings to the nation and other countries. Fifth, the study was conducted shortly after the final wave of explosive outbreaks during the concluding phase of the pandemic, following the lifting of strict control measures in China. Given the unique circumstances, generalizing the findings should be approached with caution. Also, the COVID-19 situation has been changing dramatically in China and globally. The relationships found at the ending phase of the pandemic in any country might be different from those occurring during the post-pandemic stage. Further surveillance and comparisons are warranted. Sixth, the present study did not examine factors associated with actual vaccination behaviors. This is because most participants had likely been diagnosed for less than six months and, consequently, would not yet have been eligible for COVID-19 vaccination, as the recommendation is to receive the vaccine six months after infection [22]. Furthermore, it is known that intention does not always end up in actual behavior [111]. The actual vaccination rate is expected to be much lower than that of BI-BCV. Health promotion is thus important. Seventh, the present study did not include the small number of individuals who had not taken up any dose of COVID-19 vaccines, as the focus of this study is on booster vaccination.

## Conclusions

To conclude, this is one of the few studies looking at multi-dimensional factors of intention of booster vaccination in a PCC population. It has the strength of using stratified cluster sampling that surveyed a relatively large number of participants in seven cities in different regions of China. The observed vaccination prevalence was lower than ideal, highlighting the need for targeted health promotion efforts at the community level. The findings suggest that certain cognitive factors—such as illness representation constructs (comprehensiveness) and the perceived inevitability of infection—as well as social distancing, depression, and social support, were potential determinants of BI-BCV. Notably, emotional states related to COVID-19, such as panic and emotional IR variables, did not significantly influence BI-BCV. Although the pandemic has ended, vaccination among PCC remains important. Longitudinal studies are still warranted to confirm and extend the findings.

## Supplementary Information

Supplementary Material 1.

## Data Availability

The data sets generated during and/or analyzed during the current study are available from the corresponding author on reasonable requests.

## Abbreviations

BI-BCV: Behavioral intention to take up COVID-19 booster vaccination after six months since the COVID-19 diagnosis
PCC: People having Contracted COVID-19
IR: Illness Representation
B-IPQ-COVID-19: Brief Illness Perception Questionnaire for COVID-19
PHQ-9: The 9-item Patient Health Questionnaire
CI: Confidence Interval
ORa: Adjusted Odds Ratio
ORc: Crude Odds Ratio
SD: Standard Deviation

## References

[1] Aljaberi MA, Lee K-H, Alareqe NA, Qasem MA, Alsalahi A, Abdallah AM, et al. editors. Rasch modeling and multilevel confirmatory factor analysis for the usability of the impact of event Scale-Revised (IES-R) during the COVID-19 pandemic. Healthcare: MDPI; 2022. 10.3390/healthcare10101858 PMC9602035 36292305

[2] Martinelli D, Fortunato F, Mazzilli S, Bisceglia L, Lopalco PL, Prato R. Estimating the proportion of asymptomatic COVID-19 cases in an Italian region with intermediate incidence during the first pandemic wave: an observational retrospective study. Biomed Res Int. 2022;2022(1):3401566. 35005026 10.1155/2022/3401566 PMC8733711

[3] Mohapatra RK, Tiwari R, Sarangi AK, Sharma SK, Khandia R, Saikumar G, et al. Twin combination of Omicron and Delta variants triggering a tsunami wave of ever high surges in COVID-19 cases: a challenging global threat with a special focus on the Indian Subcontinent. J Med Virol. 2022;94(5):1761–5. 35014038 10.1002/jmv.27585 PMC9015634

[4] Aljaberi MA, Al-Sharafi MA, Uzir MUH, Sabah A, Ali AM, Lee K-H, et al. editors. Psychological toll of the COVID-19 pandemic: an in-depth exploration of anxiety, depression, and insomnia and the influence of quarantine measures on daily life. Healthcare: MDPI; 2023. 10.3390/healthcare11172418 PMC10487588 37685451

[5] Organization WH. Number of COVID-19 cases reported to WHO (cumulative total) 2023 [updated December 24, 2023. Available from: https://data.who.int/dashboards/covid19/cases?n=c

[6] Nicola M, Zaid Alsafi C, Sohrabi A, Kerwan A, Al-Jabir C, Iosifidis. Maliha agha, and Riaz agha. The socio-economic implications of the coronavirus pandemic (COVID-19): A review. Int J Surg. 2020;78:185–93. 32305533 10.1016/j.ijsu.2020.04.018 PMC7162753

[7] Mohammed LA, Aljaberi MA, Amidi A, Abdulsalam R, Lin C-Y, Hamat RA, et al. Exploring factors affecting graduate students’ satisfaction toward E-learning in the era of the COVID-19 crisis. Eur J Invest Health Psychol Educ. 2022;12(8):1121–42. 10.3390/ejihpe12080079 PMC9407398 36005228

[8] Haleem A, Javaid M, Vaishya R. Effects of COVID-19 pandemic in daily life. Curr Med Res Pract. 2020;10(2):78. 32292804 10.1016/j.cmrp.2020.03.011 PMC7147210

[9] Calbi M, Langiulli N, Ferroni F, Montalti M, Kolesnikov A, Gallese V, et al. The consequences of COVID-19 on social interactions: an online study on face covering. Sci Rep. 2021;11(1):2601. 33510195 10.1038/s41598-021-81780-w PMC7844002

[10] Shah S, Diwan S, Kohan L, Rosenblum D, Gharibo C, Soin A, et al. The technological impact of COVID-19 on the future of education and health care delivery. Pain Physician. 2020;23(4S):S367. 32942794

[11] Hossain MM, Tasnim S, Sultana A, Faizah F, Mazumder H, Zou L et al. Epidemiology of mental health problems in COVID-19: a review. F1000Research. 2020;9. 10.12688/f1000research.24457.1 PMC7549174 33093946

[12] Santomauro DF, Herrera AMM, Shadid J, Zheng P, Ashbaugh C, Pigott DM, et al. Global prevalence and burden of depressive and anxiety disorders in 204 countries and territories in 2020 due to the COVID-19 pandemic. Lancet. 2021;398(10312):1700–12. 34634250 10.1016/S0140-6736(21)02143-7 PMC8500697

[13] Brooks SK, Webster RK, Smith LE, Woodland L, Wessely S, Greenberg N, et al. The psychological impact of quarantine and how to reduce it: rapid review of the evidence. Lancet. 2020;395(10227):912–20. 32112714 10.1016/S0140-6736(20)30460-8 PMC7158942

[14] Benke C, Autenrieth LK, Asselmann E, Pané-Farré CA. Lockdown, quarantine measures, and social distancing: associations with depression, anxiety and distress at the beginning of the COVID-19 pandemic among adults from Germany. Psychiatry Res. 2020;293:113462. 32987222 10.1016/j.psychres.2020.113462 PMC7500345

[15] Ma KC. Trends in Laboratory-Confirmed SARS-CoV-2 reinfections and associated hospitalizations and deaths among adults aged ≥ 18 Years—18 US jurisdictions, September 2021–December 2022. MMWR Morbidity Mortal Wkly Rep. 2023;72(25):683–9 10.15585/mmwr.mm7225a3 PMC10328471 37347715

[16] Bowe B, Xie Y, Al-Aly Z. Acute and postacute sequelae associated with SARS-CoV-2 reinfection. Nat Med. 2022;28(11):2398–405. 36357676 10.1038/s41591-022-02051-3 PMC9671810

[17] Organization WH. COVID-19 remains a persistent public health threat 2025 [cited 2025 February 9]. Available from: https://www.emro.who.int/media/news/covid-19-remains-a-persistent-public-health-threat.html

[18] Flacco ME, Acuti Martellucci C, Baccolini V, De Vito C, Renzi E, Villari P, et al. Risk of reinfection and disease after SARS-CoV‐2 primary infection: Meta‐analysis. Eur J Clin Invest. 2022;52(10):e13845. 35904405 10.1111/eci.13845 PMC9353414

[19] Hall V, Foulkes S, Insalata F, Kirwan P, Saei A, Atti A, et al. Protection against SARS-CoV-2 after Covid-19 vaccination and previous infection. N Engl J Med. 2022;386(13):1207–20. 35172051 10.1056/NEJMoa2118691 PMC8908850

[20] Hammerman A, Sergienko R, Friger M, Beckenstein T, Peretz A, Netzer D, et al. Effectiveness of the BNT162b2 vaccine after recovery from Covid-19. N Engl J Med. 2022;386(13):1221–9. 35172072 10.1056/NEJMoa2119497 PMC8908846

[21] Organization WH. Interim statement on the use of additional booster doses of Emergency Use Listed mRNA vaccines against COVID-19 2023 [cited 2023 Nov. 20]. Available from: https://www.who.int/news/item/17-05-2022-interim-statement-on-the-use-of-additional-booster-doses-of-emergency-use-listed-mrna-vaccines-against-covid-19

[22] China TSCotPsRo. Second booster shot available for at-risk people 2022 [updated December 14, 2022 cited 2023 Nov. 20]. Available from: https://english.www.gov.cn/statecouncil/ministries/202212/14/content_WS63996aa0c6d0a757729e46f7.html

[23] Staying Up to Date with COVID-19 Vaccines Centers for Disease Control and Prevention. 2024 [updated October 3, 2024; cited 2025 Jan. 4]. Available from: https://www.cdc.gov/covid/vaccines/stay-up-to-date.html?CDC_AAref_Val=https://www.cdc.gov/coronavirus/2019-ncov/vaccines/stay-up-to-date.html

[24] Abdelmoneim SA, Sallam M, Hafez DM, Elrewany E, Mousli HM, Hammad EM, et al. COVID-19 vaccine booster dose acceptance: systematic review and meta-analysis. Trop Med Infect Disease. 2022;7(10):298. 36288039 10.3390/tropicalmed7100298 PMC9611447

[25] Yu Y, Zhang X, Lau MM, Lau JT. The intention to get COVID-19 booster vaccination and its association with cognitive and emotional factors: A survey of Chinese COVID-19 infected people in Hong Kong. Vaccine. 2023. 10.1016/j.vaccine.2023.12.015 38065769

[26] Nanteer-Oteng E, Kretchy IA, Nanteer DO, Kretchy J-P, Osafo J. Hesitancy towards COVID-19 vaccination: the role of personality traits, anti-vaccine attitudes and illness perception. PLOS Global Public Health. 2022;2(12):e0001435. 36962915 10.1371/journal.pgph.0001435 PMC10021484

[27] Mora PA, McAndrew LM, Phillips A. Common-sense model of self-regulation. Encyclopedia of behavioral medicine: Springer; 2020. pp. 507 – 15.

[28] Leventhal H, Leventhal EA, Contrada RJ. Self-regulation, health, and behavior: A perceptual-cognitive approach. Psychol Health. 1998;13(4):717–33.

[29] Mo PK, Lau JT. Illness representation on H1N1 influenza and preventive behaviors in the Hong Kong general population. J Health Psychol. 2015;20(12):1523–33. 24423574 10.1177/1359105313516031

[30] Broadbent E, Ellis CJ, Thomas J, Gamble G, Petrie KJ. Further development of an illness perception intervention for myocardial infarction patients: a randomized controlled trial. J Psychosom Res. 2009;67(1):17–23. 19539814 10.1016/j.jpsychores.2008.12.001

[31] DeDonno M, Longo J, Levy X, Morris JD. Perceived susceptibility and severity of COVID-19 on prevention practices, early in the pandemic in the state of Florida. J Community Health. 2022;47(4):627–34. 35451692 10.1007/s10900-022-01090-8 PMC9024286

[32] Chen Y, Zhou R, Chen B, Chen H, Li Y, Chen Z, et al. Knowledge, perceived beliefs, and preventive behaviors related to COVID-19 among Chinese older adults: cross-sectional web-based survey. J Med Internet Res. 2020;22(12):e23729. 33293262 10.2196/23729 PMC7781588

[33] Zaid Z, Pratondo K. Public perception on COVID-19 vaccination intention. Int J Public Health Sci (IJPHS). 2021;10(4):906–13.

[34] Limbu YB, Gautam RK, Pham L. The health belief model applied to COVID-19 vaccine hesitancy: a systematic review. Vaccines. 2022;10(6):973. 35746581 10.3390/vaccines10060973 PMC9227551

[35] Janz NK, Becker MH. The health belief model: A decade later. Health Educ Q. 1984;11(1):1–47. 6392204 10.1177/109019818401100101

[36] CNN. Leaked notes from Chinese health officials estimate 250 million Covid-19 infections in December: reports 2022 [updated December 23, 2022; cited 2025 February 7]. Available from: https://edition.cnn.com/2022/12/23/china/china-covid-infections-250-million-intl-hnk/index.html

[37] Peng S, Yang T, Zhang W, Cottrell RR. Temporal changes in mental response and prevention patterns, and their impact from uncertainty stress during the transition in China from the COVID-19 epidemic to sporadic infection. Heliyon. 2023;9(8). 10.1016/j.heliyon.2023.e19090 PMC10450966 37636345

[38] Pinho S, Cruz M, Dias CC, Castro-Lopes JM, Sampaio R. Acceptance and adherence to COVID-19 Vaccination—The role of cognitive and emotional representations. Int J Environ Res Public Health. 2022;19(15):9268. 35954625 10.3390/ijerph19159268 PMC9368462

[39] Gross JJ. Emotion regulation: conceptual and empirical foundations. Handb Emot Regul. 2014;2:3–20.

[40] Iacob CI, Ionescu D, Avram E, Cojocaru D. COVID-19 pandemic worry and vaccination intention: the mediating role of the health belief model components. Front Psychol. 2021;12:674018. 34322062 10.3389/fpsyg.2021.674018 PMC8311124

[41] McCarty R. The fight-or-flight response: A cornerstone of stress research. Stress: concepts, cognition, emotion, and behavior. Elsevier; 2016. pp. 33–7.

[42] Eyllon M, Dang AP, Barnes JB, Buresh J, Peloquin GD, Hogan AC, et al. Associations between psychiatric morbidity and COVID-19 vaccine hesitancy: an analysis of electronic health records and patient survey. Psychiatry Res. 2022;307:114329. 34910966 10.1016/j.psychres.2021.114329 PMC8648380

[43] Nguyen KH, Chen S, Morris K, Chui K, Allen JD. Mental health symptoms and association with COVID-19 vaccination receipt and intention to vaccinate among adults, united States. Prev Med. 2022;154:106905. 34863815 10.1016/j.ypmed.2021.106905 PMC8634733

[44] Schomerus G, Angermeyer MC. Stigma and its impact on help-seeking for mental disorders: what do we know? Epidemiol Psychiatric Sci. 2008;17(1):31–7. 10.1017/s1121189x00002669 18444456

[45] Li F, Luo S, Mu W, Li Y, Ye L, Zheng X, et al. Effects of sources of social support and resilience on the mental health of different age groups during the COVID-19 pandemic. BMC Psychiatry. 2021;21:1–14. 33413238 10.1186/s12888-020-03012-1 PMC7789076

[46] Szkody E, Stearns M, Stanhope L, McKinney C. Stress-buffering role of social support during COVID‐19. Fam Process. 2021;60(3):1002–15. 33220082 10.1111/famp.12618 PMC7753728

[47] Jeon B, Lee H, Shon C, Kim N, Kim A, Park J et al. The association of social support with health status and health behavior among rural aged population. J Agricultural Med Community Health. 2009:13–23.

[48] Allgöwer A, Wardle J, Steptoe A. Depressive symptoms, social support, and personal health behaviors in young men and women. Health Psychol. 2001;20(3):223. 11403220

[49] Steptoe A, Wardle J, Pollard TM, Canaan L, Davies GJ. Stress, social support and health-related behavior: a study of smoking, alcohol consumption and physical exercise. J Psychosom Res. 1996;41(2):171–80. 8887830 10.1016/0022-3999(96)00095-5

[50] Jaspal R, Breakwell GM. Social support, perceived risk and the likelihood of COVID-19 testing and vaccination: cross-sectional data from the united Kingdom. Curr Psychol. 2022;41(1):492–504. 33846675 10.1007/s12144-021-01681-z PMC8026809

[51] Galanis P, Katsiroumpa A, Sourtzi P, Siskou O, Konstantakopoulou O, Katsoulas T, et al. Social support mediates the relationship between COVID-19-Related burnout and booster vaccination willingness among fully vaccinated nurses. Vaccines. 2022;11(1):46. 36679890 10.3390/vaccines11010046 PMC9861285

[52] Balasa AP. COVID–19 on lockdown, social distancing and flattening the curve–A review. Eur J Bus Manage Res. 2020;5(3):1-4.

[53] Sun KS, Lau TSM, Yeoh EK, Chung VCH, Leung YS, Yam CHK, et al. Effectiveness of different types and levels of social distancing measures: a scoping review of global evidence from earlier stage of COVID-19 pandemic. BMJ Open. 2022;12(4):e053938. 35410924 10.1136/bmjopen-2021-053938 PMC9002256

[54] Teslya A, Pham TM, Godijk NG, Kretzschmar ME, Bootsma MC, Rozhnova G. Impact of self-imposed prevention measures and short-term government-imposed social distancing on mitigating and delaying a COVID-19 epidemic: A modelling study. PLoS Med. 2020;17(7):e1003166. 32692736 10.1371/journal.pmed.1003166 PMC7373263

[55] Ainslie KE, Walters CE, Fu H, Bhatia S, Wang H, Xi X et al. Evidence of initial success for China exiting COVID-19 social distancing policy after achieving containment. Wellcome Open Res. 2020;5. 10.12688/wellcomeopenres.15843.1 PMC7236587 32500100

[56] Latkin CA, Dayton L, Yi G, Colon B, Kong X. Mask usage, social distancing, racial, and gender correlates of COVID-19 vaccine intentions among adults in the US. PLoS ONE. 2021;16(2):e0246970. 33592035 10.1371/journal.pone.0246970 PMC7886161

[57] Park J. Regional disparities in COVID-19 vaccine hesitancy: the moderating role of social distancing and vaccine rollout in the US. Int Reg Sci Rev. 2023;46(5–6):577–612.

[58] Hedlund J. Risky business: safety regulations, risk compensation, and individual behavior. Inj Prev. 2000;6(2):82–9. 10875661 10.1136/ip.6.2.82 PMC1730605

[59] China CGotPsRo. Further optimize and implement the 10 latest measures for epidemic prevention and control 2022 [updated December 7. Available from: http://www.gov.cn/xinwen/2022-12/07/content_5730470.htm

[60] Warsi JWZ. COVID rapidly spreads in China as government eases strict quarantine rules 2022 [updated December 27, 2022. Available from: https://www.pbs.org/newshour/show/covid-rapidly-spreads-in-china-as-government-eases-strict-quarantine-rules

[61] Statista. Share of people fully or partly vaccinated against coronavirus COVID-19 in China from August 2021 to June 2022 2023 [updated June 8, 2022. Available from: https://www.statista.com/statistics/1279024/china-coronavirus-covid-19-vaccination-rate/#statisticContainer

[62] Rahamim-Cohen D, Gazit S, Perez G, Nada B, Moshe SB, Mizrahi-Reuveni M et al. Survey of behaviour attitudes towards preventive measures following COVID-19 vaccination. MedRxiv. 2021:2021.04. 12.21255304.

[63] Peng X, Zhao YC, Zhu Q. Investigating user switching intention for mobile instant messaging application: taking WeChat as an example. Comput Hum Behav. 2016;64:206–16.

[64] Number of monthly active WeChat users from. 2nd quarter 2014 to 2nd quarter 2024(in millions) [Internet]. 2024 [cited November 6, 2024]. Available from: https://www.statista.com/statistics/255778/number-of-active-wechat-messenger-accounts/

[65] Zhang KFY, Cao H, Chen H, Hu T, Chen Y, Zhou X, Wang Z. Behavioral intention to receive a COVID-19 vaccination among Chinese factory workers: Cross-sectional online survey. J Med Internet Res. 2021;23(3):e24673. 33646966 10.2196/24673 PMC7945977

[66] Zhang X, Shen J, Li M, Shi Y, Wang Q, Chen F, et al. The association between socio-demographics and mental distress following COVID-19 vaccination—mediation of vaccine hesitancy. Vaccines. 2022;10(10):1697. 36298562 10.3390/vaccines10101697 PMC9611688

[67] Urrunaga-Pastor D, Bendezu-Quispe G, Herrera-Añazco P, Uyen-Cateriano A, Toro-Huamanchumo CJ, Rodriguez-Morales AJ, et al. Cross-sectional analysis of COVID-19 vaccine intention, perceptions and hesitancy across Latin America and the Caribbean. Travel Med Infect Dis. 2021;41:102059. 33848692 10.1016/j.tmaid.2021.102059 PMC8063600

[68] Yu Y, Lau JT, Lau MM, Wong MC, Chan PK. Understanding the prevalence and associated factors of behavioral intention of COVID-19 vaccination under specific scenarios combining effectiveness, safety, and cost in the Hong Kong Chinese general population. Int J Health Policy Manage. 2022;11(7):1090. 10.34172/ijhpm.2021.02 PMC9808181 33619928

[69] She R, Luo S, Lau MM, Lau JTF. The mechanisms between illness representations of COVID-19 and behavioral intention to visit hospitals for scheduled medical consultations in a Chinese general population. J Health Psychol. 2022;27(8):1846–60. 33878946 10.1177/13591053211008217

[70] Vollmann M, Salewski C. To get vaccinated, or not to get vaccinated, that is the question: illness representations about covid-19 and perceptions about covid-19 vaccination as predictors of covid-19 vaccination willingness among young adults in the Netherlands. Vaccines. 2021;9(9):941. 34579178 10.3390/vaccines9090941 PMC8473367

[71] Na Zhang RF, Inda Soong,Karen KK, Chan. Conrad lee, Alice ng, wing kin sze, Janice tsang, victor lee, Wendy wing Tak lam psychometric assessment of the Chinese version of the brief illness perception questionnaire in breast cancer survivors. PLoS ONE. 2017;12(3):e0174093. 28319160 10.1371/journal.pone.0174093 PMC5358881

[72] Yu YLR, Ng JH-Y, Lau MMC, Ip TKM, Lau JTF. Illness representation of COVID-19 affected public’s support and anticipated panic regarding the living with the virus policy: a cross-sectional study in a Chinese general population. Front Public Health. 2023;11:1158096. 10.3389/fpubh.2023.1158096 PMC10506401 37727606

[73] Fitzpatrick KM, Drawve G, Harris C. Facing new fears during the COVID-19 pandemic: the state of america’s mental health. J Anxiety Disord. 2020;75:102291. 32827869 10.1016/j.janxdis.2020.102291 PMC7425672

[74] Mertens G, Gerritsen L, Duijndam S, Salemink E, Engelhard IM. Fear of the coronavirus (COVID-19): predictors in an online study conducted in March 2020. J Anxiety Disord. 2020;74:102258. 32569905 10.1016/j.janxdis.2020.102258 PMC7286280

[75] Nelson LM, Simard JF, Oluyomi A, Nava V, Rosas LG, Bondy M, et al. US public concerns about the COVID-19 pandemic from results of a survey given via social media. JAMA Intern Med. 2020;180(7):1020–2. 32259192 10.1001/jamainternmed.2020.1369 PMC7139509

[76] Tan Y, Lin X, Wu D, Chen H, Jiang Y, He T, et al. Different trajectories of panic and the associated factors among unmarried Chinese during the COVID-19 pandemic. Appl Psychology: Health Well‐Being. 2020;12(4):967–82. 33016617 10.1111/aphw.12238 PMC7675528

[77] Li J, Mo PK, Wu AM, Lau JT. Roles of self-stigma, social support, and positive and negative affects as determinants of depressive symptoms among HIV infected men who have sex with men in China. AIDS Behav. 2017;21:261–73. 26896120 10.1007/s10461-016-1321-1 PMC4992470

[78] Yu Y, Lau JT, Lau MM. Development and validation of the conservation of resources scale for COVID-19 in the Chinese adult general population. Curr Psychol. 2023;42(8):6447–56. 34155427 10.1007/s12144-021-01933-y PMC8210506

[79] Kroenke K, Spitzer RL, Williams JB. The PHQ-9: validity of a brief depression severity measure. J Gen Intern Med. 2001;16(9):606–13. 11556941 10.1046/j.1525-1497.2001.016009606.x PMC1495268

[80] Wang W, Bian Q, Zhao Y, Li X, Wang W, Du J, et al. Reliability and validity of the Chinese version of the patient health questionnaire (PHQ-9) in the general population. Gen Hosp Psychiatry. 2014;36(5):539–44. 25023953 10.1016/j.genhosppsych.2014.05.021

[81] Zhang WYX, Zhao J, Yang F, Jia Y, Cui C, Yang X. Depression and Psychological-Behavioral responses among the general public in China during the early stages of the COVID-19 pandemic: survey study. J Med Internet Res. 2020;22(9):e22227. 32886066 10.2196/22227 PMC7501583

[82] Yu Y, Lau JTF, Lau MM. Levels and factors of social and physical distancing based on the theory of planned behavior during the COVID-19 pandemic among Chinese adults. Translational Behav Med. 2021;11(5):1179–86. 10.1093/tbm/ibaa146 PMC7928604 33598679

[83] Wong LP, Alias H, Siaw Y-L, Muslimin M, Lai LL, Lin Y, et al. Intention to receive a COVID-19 vaccine booster dose and associated factors in Malaysia. Hum Vaccines Immunotherapeutics. 2022;18(5):2078634. 10.1080/21645515.2022.2078634 PMC9481074 35648441

[84] Banik R, Islam MS, Pranta MUR, Rahman QM, Rahman M, Pardhan S, et al. Understanding the determinants of COVID-19 vaccination intention and willingness to pay: findings from a population-based survey in Bangladesh. BMC Infect Dis. 2021;21(1):892. 34465297 10.1186/s12879-021-06406-y PMC8406014

[85] Cordina M, Lauri MA. Attitudes towards COVID-19 vaccination, vaccine hesitancy and intention to take the vaccine. Pharm Pract (Granada). 2021;19(1). 10.18549/PharmPract.2021.1.2317 PMC8005329 33828623

[86] Leng A, Maitland E, Wang S, Nicholas S, Liu R, Wang J. Individual preferences for COVID-19 vaccination in China. Vaccine. 2021;39(2):247–54. 33328140 10.1016/j.vaccine.2020.12.009 PMC7719001

[87] Siu JY-m, Cao Y, Shum DH. Perceptions of and hesitancy toward COVID-19 vaccination in older Chinese adults in Hong kong: A qualitative study. BMC Geriatr. 2022;22(1):1–16. 35387602 10.1186/s12877-022-03000-y PMC8985396

[88] Aklil MB, Temesgan WZ, Gessesse DN, Kassa BG, Tiguh AE, Kebede AA, et al. editors. Willingness to receive COVID-19 vaccine and associated factors among college students in Gondar city, Northwest ethiopia. Frontiers in education. Frontiers Media SA; 2022.

[89] Al-Mohaithef M, Padhi BK. Determinants of COVID-19 vaccine acceptance in Saudi arabia: a web-based National survey. J Multidisciplinary Healthc. 2020;13:1657–63. 10.2147/JMDH.S276771 PMC7686470 33262600

[90] Wu J, Shen Z, Li Q, Tarimo CS, Wang M, Gu J, et al. How urban versus rural residency relates to COVID-19 vaccine hesitancy: A large-scale National Chinese study. Soc Sci Med. 2023;320:115695. 36736053 10.1016/j.socscimed.2023.115695 PMC9846885

[91] Ma R, Suo L, Lu L, Pang X. Willingness of the general public to receive the COVID-19 vaccine during a second-level alert—Beijing municipality, china, May 2020. China CDC Wkly. 2021;3(25):531. 34594928 10.46234/ccdcw2021.118 PMC8393022

[92] Wu J, Li Q, Silver Tarimo C, Wang M, Gu J, Wei W, et al. COVID-19 vaccine hesitancy among Chinese population: a large-scale National study. Front Immunol. 2021;12:781161. 34912346 10.3389/fimmu.2021.781161 PMC8666422

[93] Schulman-Green D, Jaser SS, Park C, Whittemore R. A metasynthesis of factors affecting self‐management of chronic illness. J Adv Nurs. 2016;72(7):1469–89. 26781649 10.1111/jan.12902 PMC4891247

[94] Wang Z, Fang Y, Dong W, Lau M, Mo PK. Illness representations on pneumonia and Pneumococcal vaccination uptake among community-living Chinese people with high-risk conditions aged ≥ 65 years—a population-based study. Hum Vaccines Immunotherapeutics. 2021;17(5):1455–62. 10.1080/21645515.2020.1814653 PMC8078741 32991245

[95] Setbon M, Raude J. Factors in vaccination intention against the pandemic influenza A/H1N1. Eur J Pub Health. 2010;20(5):490–4. 20444821 10.1093/eurpub/ckq054 PMC7314046

[96] Sherman KA, Kilby CJ, Moore DM, Shaw L-K. The importance of coherently Understanding cervical cancer vaccination: factors associated with young Australian women’s uptake of the HPV vaccine. Health Psychol Behav Med. 2017;5(1):358–71.

[97] Maykrantz SA, Gong T, Petrolino AV, Nobiling BD, Houghton JD. How trust in information sources influences preventative measures compliance during the COVID-19 pandemic. Int J Environ Res Public Health. 2021;18(11):5867. 34070713 10.3390/ijerph18115867 PMC8198292

[98] Wang P-W, Ahorsu DK, Lin C-Y, Chen I-H, Yen C-F, Kuo Y-J, et al. Motivation to have COVID-19 vaccination explained using an extended protection motivation theory among university students in china: the role of information sources. Vaccines. 2021;9(4):380. 33924604 10.3390/vaccines9040380 PMC8070343

[99] Zheng H, Jiang S, Wu Q. Factors influencing COVID-19 vaccination intention: the roles of vaccine knowledge, vaccine risk perception, and doctor-patient communication. Patient Educ Couns. 2022;105(2):277–83. 34565643 10.1016/j.pec.2021.09.023 PMC8450210

[100] Wong LP, Alias H, Wong P-F, Lee HY, AbuBakar S. The use of the health belief model to assess predictors of intent to receive the COVID-19 vaccine and willingness to pay. Hum Vaccines Immunotherapeutics. 2020;16(9):2204–14. 10.1080/21645515.2020.1790279 PMC7553708 32730103

[101] Rogers RW. A protection motivation theory of fear appeals and attitude Change1. J Psychol. 1975;91(1):93–114. 28136248 10.1080/00223980.1975.9915803

[102] Ruiter RAC, Kessels LTE, Peters GJY, Kok G. Sixty years of fear appeal research: current state of the evidence. Int J Psychol. 2014;49(2):63-70. 10.1002/ijop.12042 24811876

[103] Cokro F. Supporting and inhibiting factors of accepting COVID-19 booster vaccination in the elderly in North jakarta, Indonesia. Pharm Pract. 2022;20(4):1–9. 10.18549/PharmPract.2022.4.2748 PMC9891777 36793907

[104] Lazarus RS, Folkman S. Stress, appraisal, and coping. Springer publishing company; 1984.

[105] Resna RW, Amanda T, Nofiantoro W, Mustajidah M, Ashbahna DM, Iskandar R, et al. Family support for COVID-19 vaccination in older adults: scoping review. J Nurs Sci Update (JNSU). 2023;11(1):90–9.

[106] Ajzen I. The theory of planned behavior. Organ Behav Hum Decis Process. 1991;50(2):179–211.

[107] Ariful Kabir K, Tanimoto J. Assessing the instantaneous social dilemma on social distancing attitudes and vaccine behavior in disease control. Sci Rep. 2024;14(1):14244. 38902279 10.1038/s41598-024-64143-z PMC11190193

[108] Saha S, Samanta G, Nieto JJ. Impact of optimal vaccination and social distancing on COVID-19 pandemic. Math Comput Simul. 2022;200:285–314. 35531464 10.1016/j.matcom.2022.04.025 PMC9056068

[109] Wright L, Steptoe A, Mak HW, Fancourt D. Do people reduce compliance with COVID-19 guidelines following vaccination? A longitudinal analysis of matched UK adults. J Epidemiol Community Health. 2022;76(2):109–15. 34244309 10.1136/jech-2021-217179 PMC8275358

[110] Jia JS, Yuan Y, Jia J, Christakis N. Risk perception and behavior change after personal vaccination for COVID-19 in the USA. 2022.

[111] Wang J, Zhu H, Lai X, Zhang H, Huang Y, Feng H, et al. From COVID-19 vaccination intention to actual vaccine uptake: A longitudinal study among Chinese adults after six months of a National vaccination campaign. Expert Rev Vaccines. 2022;21(3):385–95. 34974779 10.1080/14760584.2022.2021076

