# Supplementary material for: Multi-dimensional factors of behavioral intention of COVID-19 booster vaccination among people having contracted COVID-19—a 7-city study conducted during the last national major outbreak in China
Source: BMC Public Health. 2025 Dec 30;25:4391. doi: 10.1186/s12889-025-23915-6 (PMC12754956; doi:10.1186/s12889-025-23915-6)
Supplement: Supplementary file 1 — Supplementary Material 1. [file 12889_2025_23915_MOESM1_ESM.docx]

**Questionnaire**

Dear friends,

Thank you very much for participating in this survey. This is a collaborative project between Zhejiang University School of Public Health, Sun Yat-sen University School of Public Health, Fudan University School of Public Health, Wenzhou Medical University, and Peking University School of Psychology and Cognitive Sciences, in conjunction with the Centers for Disease Control and Prevention. The purpose of this survey is to understand people’s perspectives on COVID-19 pandemic and its impact, so as to provide certain reference suggestions for the management and response of this public health event. We sincerely hope that you can take some time to support this investigation.

There are no right or wrong answers to the questions. Please answer according to the true situation. Participation in the survey is voluntary and anonymous. You do not need to provide your name, address, WeChat ID or any personal information. All information collected will only be used for research purposes and will be kept strictly confidential. Only one member of your household needs to fill it out. Thank you!

□1 I am aware of the above situation and voluntarily participate in this survey

□2 I do not participate in this survey (termination of survey)

**You are 18 years old or above: □No (termination of survey) □Yes**

1. **Infection**
2. Have you ever been infected with COVID-19 (i.e. had a positive antigen/nucleic acid test)?

**□**1 No (skip to the end) □2 Positive antigen test □3 Positive nucleic acid test

b. The date you discovered the positive result:

□1 Year 2020 □2 Year 2021 □3 January to November 2022 □4 December 1, 2022, or later (please specify the date______)

c. Have you ever received COVID-19 vaccine?

□1 No

□2 Took one dose (skip to d)

□3 Took doses (skip to d)

□4 Took three doses (skip to d)

□5 Took four doses (skip to d)

d. How likely do you plan to take up the COVID-19 vaccine after six months from now?

□1 Definitely not □2 Probably not □3 Half and half □4 Probably yes □5 Definitely yes

**Perceptions**

| 2. B-IPQ | |
| --- | --- |
| 1. How much has the COVID-19 infection affected your life? | Does not affect at all Severely affects my life  0 1 2 3 4 5 6 7 8 9 10 |
| 1. How long do you think your COVID-19 condition would last? | A very short time Forever  0 1 2 3 4 5 6 7 8 9 10 |
| 1. How much control do you have over your COVID-19 conditions? | Absolutely no control Extreme amount of control  0 1 2 3 4 5 6 7 8 9 10 |
| 1. How much do you think treatment can help your COVID-19 conditions? | Not at all Extremely helpful  0 1 2 3 4 5 6 7 8 9 10 |
| 1. How many symptoms related to COVID-19 have you experienced? | No symptoms at all Many severe symptoms 0 1 2 3 4 5 6 7 8 9 10 |
| 1. How concerned are you about your COVID-19 conditions? | Not at all concerned Extremely concerned 0 1 2 3 4 5 6 7 8 9 10 |
| 1. How well do you feel you understand COVID-19? | Not understand at all Understand very well  0 1 2 3 4 5 6 7 8 9 10 |
| 1. How much have your COVID-19 conditions affected you emotionally (e.g., make you angry, scared, upset, or depressed)? | Not at all affected Extremely affected  0 1 2 3 4 5 6 7 8 9 10 |

| 3. Perceived inevitable infection | Strongly disagree | Disagree | Neutral | Agree | Strongly agree |
| --- | --- | --- | --- | --- | --- |
| 1. I would be unable to protect myself from contracting/re-contracting COVID-19, no matter what protection measures I am going to take up | 1 | 2 | 3 | 4 | 5 |

**Behaviours**

| **4.** In the past week, how often did you do the following | Never | Seldomly | Sometimes | Frequently | Always |
| --- | --- | --- | --- | --- | --- |
| 1. Not going out unless necessary | 1 | 2 | 3 | 4 | 5 |
| 1. Avoiding attending social gatherings | 1 | 2 | 3 | 4 | 5 |
| 1. Reducing meeting and contacting people you know | 1 | 2 | 3 | 4 | 5 |
| 1. Avoiding going to crowded places | 1 | 2 | 3 | 4 | 5 |
| 1. Avoiding taking public transportation | 1 | 2 | 3 | 4 | 5 |

**Psychosocial factors**

| **5.** Social support | Strongly disagree | Disagree | Somewhat disagree | Neutral | Somewhat agree | Agree | Strongly agree |
| --- | --- | --- | --- | --- | --- | --- | --- |
| 1. a. Currently if you need someone to talk to or for emotional support, your family and friends will be there for you | 1 | 2 | 3 | 4 | 5 | 6 | 7 |
| 1. b. If you need instrumental help (such as financial assistance or facing life difficulties), your family and friends will provide you with sufficient support | 1. 1 | 2 | 3 | 4 | 5 | 6 | 7 |
| 1. c. If you or your family members need COVID-19-related medicine (such as antipyretics) and antigen testing kits, you can get them from your relatives and friends | 1 | 2 | 3 | 4 | 5 | 6 | 7 |

6. Do you feel concerned or panicked due to any of the following situations? (Multiple selections allowed)

□1 Panic about older people or children in your family being infected or re-infected with COVID-19

□2 Panic about lack of medicine for COVID-19 or antigen testing kits, and

□3 Panic about the COVID-19 situation in the city you are living in

□4 None of the above

| 7. Over the last two weeks, how often have you been bothered by any of the following problems (PHQ-9): | Not at all | Several days | More than half the days | Nearly every day |
| --- | --- | --- | --- | --- |
| a. Little interest or pleasure in doing things | 0 | 1 | 2 | 3 |
| b. Feeling down, depressed, or hopeless | 0 | 1 | 2 | 3 |
| c. Trouble falling or staying asleep, or sleeping too much | 0 | 1 | 2 | 3 |
| d. Feeling tired or having little energy | 0 | 1 | 2 | 3 |
| e. Poor appetite or overeating | 0 | 1 | 2 | 3 |
| f. Feeling bad about yourself—or that you are a failure or have let yourself or your family down | 0 | 1 | 2 | 3 |
| g. Trouble concentrating on things, such as reading the newspaper or watching television? | 0 | 1 | 2 | 3 |
| h. Moving or speaking so slowly that other people could have noticed. Or, the opposite—being so fidgety or restless that you have been moving around a lot more than usual | 0 | 1 | 2 | 3 |
| i. Thoughts that you would be better off dead, or of hurting yourself in some way | 0 | 1 | 2 | 3 |

Basic information:

8. Sex： □1 Male □2 Female

9. Age： ______years

10. What is your occupation? □1 Full-time employment □2 Part-time employment □3 Retired □4 Homemaker □5 Unemployed □6 Student □7 Other:___

11. What is your current marital status? □1 Single □2 Married □3 Separated/divorced □4 Widowed □5 Cohabiting □6 Other, please specify________

12. Your highest education level:

□1. Junior high school/secondary vocational school or below □2. High school/higher vocational school □3. College □4. Bachelor’s degree □5. Master’s degree □6. Doctoral degree

13. Do you have any chronic diseases (such as hypertension, diabetes, chronic lung disease, myocardial infarction, heart failure, cerebrovascular disease, neurodegenerative diseases, ulcerative diseases such as gastric ulcer, liver disease, and tumors)?

□1 None □2 Yes, poorly controlled □3 Yes, moderately controlled □4 Yes, well controlled

14. What city do you live in?

□1 Shanghai □2 Neijiang □3 Guangzhou □4 Hangzhou □5 Jinan □6 Shihezi □7 Mianyang

15. What type of community are you living in

□1 Urban □2 Rural
